# Celastrol ameliorates vascular neointimal hyperplasia through Wnt5a-involved autophagy

**DOI:** 10.7150/ijbs.58715

**Published:** 2021-06-22

**Authors:** Ya-Ning Shi, Le-Ping Liu, Chang-Feng Deng, Tan-Jun Zhao, Zhe Shi, Jian-Ye Yan, Yong-Zhen Gong, Duan-Fang Liao, Li Qin

**Affiliations:** 1 School of Pharmacy, Hunan University of Chinese Medicine, Changsha, Hunan, China.; 2 Division of Stem Cell Regulation and Application, Hunan University of Chinese Medicine, Changsha, Hunan, China.; 3 Institue of Innovation and Applied Research in Chinese Medicine, Hunan University of Chinese Medicine, Changsha, Hunan, China.

**Keywords:** celastrol, vascular smooth muscle cells, neointimal formation, autophagy, restenosis

## Abstract

Neointimal hyperplasia caused by the excessive proliferation of vascular smooth muscle cells (VSMCs) is the pathological basis of restenosis. However, there are few effective strategies to prevent restenosis. Celastrol, a pentacyclic triterpene, has been recently documented to be beneficial to certain cardiovascular diseases. Based on its significant effect on autophagy, we proposed that celastrol could attenuate restenosis through enhancing autophagy of VSMCs. In the present study, we found that celastrol effectively inhibited the intimal hyperplasia and hyperproliferation of VSMCs by inducing autophagy. It was revealed that autophagy promoted by celastrol could induce the lysosomal degradation of c-MYC, which might be a possible mechanism contributing to the reduction of VSMCs proliferation. The Wnt5a/PKC/mTOR signaling pathway was found to be an underlying mechanism for celastrol to induce autophagy and inhibit the VSMCs proliferation. These observations indicate that celastrol may be a novel drug with a great potential to prevent restenosis.

## Introduction

Cardiovascular diseases (CVDs) are the leading cause of death assessed by the Global Burden of Disease (GBD) study 1. A variety of effective therapies, such as percutaneous coronary intervention (PCI), percutaneous transluminal coronary angioplasty (PTCA), stent implantation and bypass surgery, play an active role in the prevention and treatment of CVDs 2-4. Although the safety and effectiveness of coronary stent devices are improved, postoperative restenosis and device thrombosis remain the most prominent pathological problem and may lead to poor clinical outcomes 5. As reported in a trial with 1713 patients enrolled, the cumulative 5-year risk of at least moderate restenosis (≥50%) was 40.7% in stenting cases and 29.6% in endarterectomy cases 6. Restenosis is the development of lumen stenosis caused by mechanical vessel injury. Therefore, inhibition of restenosis is crucial to ameliorate the patient outcomes.

Vascular remodeling and intimal hyperplasia are the main pathological causes of restenosis. During the development of restenosis, vascular smooth muscle cells (VSMCs) undergo phenotypic transformation from a contractile state to a proliferative state and migrate from the middle layer to intima 7. As one of the most potent mitogen, platelet-derived growth factor-BB (PDGF-BB) is highly effective in regulating phenotype transformation (contractile-to-synthetic) of VSMCs 8, 9. PDGF-BB released by vascular injury can *in situ* induce VSMCs proliferation, migration and even intimal hyperplasia 10.

Autophagy, a life-sustaining cellular catabolic process, is mainly characterized by emergence of double membrane vesicles (including autophagosomes and autophagolysosomes) and is usually observed when a large number of cells need to be eliminated or phagocytes are inaccessible into dying cells 11. During the process of autophagy, the fusion of autophagosomes and lysosomes generates autophagolysosomes for degradation. Growing evidence suggests that autophagy plays a crucial regulatory role in maintaining VSMCs homeostasis and the pathogenesis of vascular remodeling of restenosis 12, 13. Of note is that enhanced autophagy of VSMCs can prevent excessive proliferation and reduce intimal hyperplasia 14, 15. In contrast, autophagy defects in VSMCs accelerate migration and promote neointimal formation as well as the development of atherosclerosis 16-18. Therefore, it is reasonable to speculate that inhibition of VSMCs overgrowth by regulating autophagy might be a potential treatment strategy for restenosis.

Celastrol (CeT), a pentacyclic triterpene, is one of the key bioactive components purified from the roots of *Tripterygium wilfordii* (thunder god vine) and has been documented to be a leptin sensitizer for the treatment of dietary obesity 19. Recently, celastrol was found as a new drug for the treatment of cardiovascular diseases 20, 21. There is growing evidence that celastrol exerts anti-atherosclerosis effects by regulating lipid metabolism, reducing oxidative stress, and improving vascular function, *etc* 22-24. In addition, celastrol inhibits inflammation response and prevents the development of cancers (glioma, non-small cell lung cancer, osteosarcoma, *etc*.) by inducing autophagy 25-28. However, the effect of celastrol on vascular neointimal hyperplasia has not yet been studied. Since autophagy is involved in the proliferation of VSMCs, we proposed that celastrol may inhibit the proliferation of VSMCs and intimal hyperplasia, which may further provide an insight for implementing novel therapeutic strategies to prevent restenosis.

## Materials and Methods

### Materials

Dulbecco's modified Eagle's medium (DMEM), fetal bovine serum (FBS), and trypsin-EDTA were purchased from Gibco BRL (Carlsbad, CA, USA). Celastrol (C0869), chloroquine (CQ) (C6628), phorbol ester (PMA, P1585), MHY-1485 (SML0810) and 4′,6-diamidino-2-phenylindole (DAPI) were the products of Sigma-Aldrich (St. Louis, MO, USA). Recombinant human PDGF-BB (100-14B) was obtained from Peprotech (Rocky Hill, NJ, USA). Lentiviral particles and plasmids were purchased from Invitrogen (CA, USA). Cell count kit-8 (CCK-8) (C0038) and anti-α-Tubulin (AF0001) antibody were obtained from Beyotime Institute of Biotechnology (Shanghai, China). The 5-Ethynyl-2'-deoxyuridine (EdU) kit (C10310-1) was obtained from RiboBio (Guangzhou, China). An adenovirus expressing monomeric red fluorescent protein-green fluorescent protein-LC3 (mRFP-GFP-LC3) (HB-AP210 0001) was provided by HanBio Technology Co. Ltd. (Shanghai, China). Antibodies against microtubule-associated protein 1 light chain 3 (LC3) A/B (ab128025), sequestosome 1 (SQSTM1, best known as p62) (ab155686), c-MYC (ab32072), Wnt5a (ab72583), protein kinase C (PKC, ab179521), p-PKC (ab75837), mammalian target of rapamycin (mTOR, ab2732), p-mTOR (ab109268), goat anti-rabbit IgG H&L (Alexa Fluor® 488) (ab150077), and donkey anti-mouse IgG H&L (Alexa Fluor® 594) (ab150108) were purchased from Abcam (Cambridge, MA, USA). The peroxidase‐conjugated anti‐rabbit (SA00001-2) and anti-mouse (SA00001-1) secondary antibodies were purchased from Proteintech (Chicago, Illinois, USA). Polyvinylidene difluoride (PVDF) membrane and enhanced chemiluminescence (ECL) plus kit (WBKLS0500) were provided by Millipore Corporation (Billerica, MA, USA). Adenovirus (U6/CMV-GFP&PURO)-mediated Wnt5a-specific small interfering RNA (siWnt5a) (forward: 5'-GGUUGUUAUAGAAGCUAAUTT-3', reverse: 5'-AUUAGCUUCUAUAACAACCTT-3') was produced by GenePharma (Shanghai, China). The co-immunoprecipitation (Co-IP) (26149) kit was provided by Thermo Fisher Scientific (Waltham, MA, USA). HRP-conjugated secondary antibody and DAB peroxidase substrate solution (PV-9000) were purchased from ZSGB-BIO (Beijing, China).

### Animals

Eight-week-old male C57BL/6 mice (Slac Laboratory Animal Co. Ltd., Shanghai, China) were used in this study. All procedures were performed according to the Guide for the Care and Use of Laboratory Animals published by the US National Institutes of Health (NIH Publication, 8th Edition, 2011). All experiments were approved by the Animal Ethics Committee of the Hunan University of Chinese Medicine (Hunan, China). Mice were maintained in a 12-h dark-light cycle and had free access to diet and water.

### Mouse femoral artery injury model

The procedure performed for the mouse model was similar to the one described earlier 29. C57BL/6 mice were anesthetized by inhalation of isoflurane. Under a surgical microscope, a groin incision was formed by using microscopic instruments. An arteriotomy was operated in femoral arteries. A straight spring wire (0.38 mm in diameter; Cook, Bloomington, IN, USA) was inserted into the femoral artery to the bifurcation of the abdominal aorta. The guide wire was carefully pulled three times back and forth to result in intravascular injury. After removing the guide wire, the femoral artery was ligated. Sham-operated arteries used as control were dissected without the procedure of guide wire pulling. Celastrol was dissolved in pure DMSO and diluted with PBS to a final concentration of 1% DMSO for intraperitoneal injection. Adenovirus-mediated siWnt5a was dissolved in PBS for tail vein injection and local infiltration. After the injury, the mice were randomly allocated to experiment groups. To evaluate the effect of celastrol on intimal hyperplasia and VSMCs autophagy *in vivo*, the mice were divided into seven groups. Group 1 was untreated as control (Sham). Group 2 underwent surgery (Model). Group 3 received only vehicle (DMSO) after the injury. Group 4, group 5, and group 6 received intraperitoneal injection of celastrol at 0.5, 1, 2 mg/kg/day respectively for 4 weeks immediately after surgery. To evaluate how Wnt5a knockdown affect neointimal formation and VSMCs autophagy *in vivo*, the mice were divided into five groups. Group 1 received no treatment to serve as control. Group 2 underwent surgery. Group 3 was given intraperitoneal injection of 2 mg/kg/day celastrol for 2 weeks immediately after surgery. Group 4 received immediately tail vein injection of 6 × 10^9^ PFU adenovirus-mediated siWnt5a and local infiltration of 2 × 10^9^ PFU adenovirus-mediated siWnt5a for 2 weeks immediately after surgery. Group 5 received tail vein injection of 6 × 10^9^ PFU adenovirus vector and local infiltration of 2 × 10^9^ PFU adenovirus vector for 2 weeks immediately after surgery. The arteries were collected on 14th or 28th days after the surgery for transmission electron microscopy (TEM) observation, hematoxylin-eosin (HE)-staining, western blot analysis and immunohistochemistry.

### Histologic examination

Two weeks or four weeks after operation, the control and injured femoral arteries were fixed in 4% paraformaldehyde for 24 h and then embedded in paraffin. The paraffin cross sections were stained with hematoxylin-eosin. The intimal area was determined by subtraction of the lumen area from the internal elastic lamina (IEL) area (i.e., intimal area=IEL area - lumen area). The medial area was calculated by subtraction of the IEL area from the external elastic lamina (EEL) area (i.e., medial area=EEL area - IEL area). For morphologic analysis of neointimal formation, a professional image analysis software Image-Pro Plus 6.0 was used to measure the intima/media ratios by tracing the areas of the EEL, IEL, and lumen area. The intima/media ratios were obtained with Area_intima_ devided by Area_media_.

### Cell culture

Human vascular smooth muscle cells (obtained from Sun yat-sen university, Guangzhou, China) maintained in DMEM supplemented with 10% FBS in a humidified 5% CO_2_ and 95% air atmosphere at 37˚C.

### Plasmids and lentivirus preparation

A stable Wnt5a-overexpression VSMCs line (VSMC-Wnt5a) was constructed by lentivirus transfection as described previously 30. Lentiviral particles were packaged and used for cell transduction according to the manufacturer's instructions. Lentivirus was prepared with co-transfection of 293T cells with plasmids psPAX2, pMD2.G and pLenti6/V5-Wnt5a or pLenti6/V5 using Lipofectamine 2000 according to the manufacturer's protocol. Lentivirus-containing media were collected from plates at 24 h and 48 h after transfection. Then, VSMCs were incubated with lentivirus-containing media and 8 mg/mL polybrene for 24 h. All survived cells were selected by 5 μg/mL blasticidin.

### CCK-8 assay

The proliferation of VSMCs was measured by using CCK-8 assay kit. Approximately 3 × 10^3^ VSMCs in 100 μL medium were cultured in triplicate in 96-well plates. The CCK-8 reagent (10 μL) was added to the well, and then, the cells were incubated in a 5% CO_2_ atmosphere at 37°C for 30 min. The optical density (OD) value at 450 nm was measured using a microplate reader (SYNERGYH4, BioTek, Winooski, VT, USA). Cell viability (%) = (OD_experimental group_ - OD_blank group_)/(OD_control group_ - OD_blank group_) × 100%

### EdU assay

The EdU assay kit was used to detect cell proliferation. Approximately 3 × 10^3^/well VSMCs were cultured in 96-well plates for 24 h. Firstly, the cells were incubated with 50 μM EdU for 2 h at 37 °C, and then fixed in 4% paraformaldehyde for 0.5 h. Glycine was utilized to neutralize 4% paraformaldehyde and 0.5% TritonX-100 was used to permeabilize. Subsequently, the cells were incubated with Click-iT EdU reaction solution for 0.5 h. Finally, Hoechst 33342 (100 μL) was used to stain the nuclei. The image of EdU-positive cells was captured using an inverted fluorescence microscope (Olympus, Tokyo, Japan). EdU-positive cells ratio (%) = EdU-positive cells/total cells × 100%

### Wound-healing assay

When the confluence of VSMCs reached 80%, a 200 μL pipette tip was used to form a wound in the confluent cells. The cells were then rinsed with medium to remove any free floating cells and debris. The medium was added and the plates were incubated at 37°C. We observed different stages of wound healing along the scratch line and photographed the representative scratch lines. Each experiment was repeated three times. Average value of scratch width = scratch area/scratch length. Migration rate = (scratch width of 0 h - scratch width of 6 h, 12 h or 24 h)/scratch width of 0 h × 100%

### Transmission electron microscopy

VSMCs or femoral arteries were flushed with PBS twice. First, the cells or femoral arteries were fixed with 2.5% glutaraldehyde at 4˚C for 2 h, and washed with 0.1 M phosphate buffer for at least 0.5 h. Second, the cells or femoral arteries were fixed in 0.1 M sodium phosphate buffer containing 1% osmium tetroxide for 2 h at 4˚C. Afterwards, ethanol dehydration, embedding, polymerization, slicing and staining were performed. Finally, the 50-70 nm flakes were stained for 15 min with uranyl acetate and citric acid, respectively. Images were obtained using a Hitachi HT7700 TEM.

### Autophagic flux assessment using autophagic double-labeled adenovirus

To detect and analyze the autophagic flux, VSMCs were transfected with adenovirus-mediated tandem fluorescent mRFP-GFP-tagged LC3. VSMCs were seeded onto 24-well plates and allowed to achieve 50% confluent upon transfection. Adenovirus infection was performed according to the manufacturer's instructions. VSMCs were incubated with adenoviruses at multiplicities of infection (MOI) of 100 for 4 h at 37°C in the medium and then adenoviruses were removed. VSMCs continued to grow in the medium for 24 h at 37°C. Thereafter, the cells were incubated in a medium containing 200 nM celastrol and/or other reagents (including 10 ng/mL PDGF-BB and 10 μM CQ) for 24 h. Finally, autophagy was observed under confocal laser scanning microscope (Leica, TCS SP8, Germany).

### Western blot analysis

VSMCs or femoral arteries were homogenized in radio-immunoprecipitation assay (RIPA) lysis buffer containing phenylmethanesulfonyl fluoride (PMSF). The cell lysate was centrifuged at 12,000 g for 10~20 min at 4℃. The total proteins in the cell lysate were quantified using a bicinchoninic acid (BCA) assay kit, and then separated by 8%-12% sodium dodecyl sulfate-polyacrylamide gel electrophoresis (SDS-PAGE) and transferred to PVDF membranes. After blocking with 5% skimmed milk for 2 h, PVDF membranes were incubated with a primary antibody overnight at 4°C, and then incubated with a secondary antibody for 2 h. The specific proteins were visualized with ECL plus kit using visualizer (Tanon-5200, Shanghai, China), and band intensity was measured as integrated optical density (IOD).

### Co-IP and Mass Spectrometry (MS) analysis

Co-IP was carried out using Co-IP Kit, following the manufacturer's instructions with minor modifications. In brief, 10 μg of anti-Wnt5a antibody or IgG antibody was bound to AminoLink® Plus Coupling Resin. The VSMCs were lysed with 500 μL of ice-cold IP lysis/wash buffer. The lysates of celastrol-treated VSMCs or celastrol were incubated with antibody-coupled resin at 4°C overnight. The resin was washed five times with IP lysis/wash buffer and then eluted with 60 μL of elution buffer. The eluted Wnt5a proteins and celastrol were subjected to western blot or MS analysis.

MS was performed with an electrospray source (ESI) in the positive mode, and the data were collected in MS continuum mode. Analysis was performed in full scan mode. The scan time was 0.2 s, and scan range was from 100 to 1500 m/z. Capillary and cone voltage were set at 3.0 kV and 40 V respectively. Source temperature was set to 120℃. Desolvation gas was set to 800 L/h at a temperature of 400℃. Cone gas was set to 50 L/h. MCP detector voltage was set at 2785 V. An alternation low-energy (collision cell energy of 6 V) and elevated energy (collision cell energy ramped from 20 V to 30 V) were set to acquire the parent ions and their fragmentation data.

### Immunofluorescence assays

VSMCs were incubated on glass coverslips in a 24-well plate for 24 h and then incubated with 200 nM celastrol and/or other reagents (including 10 ng/mL PDGF-BB and 10 μM CQ) for 24 h. The medium was removed and the cells were washed with PBS thrice. The cells were then fixed with 4% paraformaldehyde for 0.5 h. The 4% paraformaldehyde was removed and the cells were permeabilized with 0.5% Triton X-100 in PBS for 20 minutes, then washed three times with PBS. Next, the cells were blocked with 3% BSA in PBS for 2 h at room temperature. The p62 protein was detected with the p62 antibody (working dilution 1:500) at 25℃ for 1 h. The cells were washed three times with PBS and incubated with a secondary antibody (goat anti-rabbit IgG H&L (Alexa Fluor® 488); working dilution 1:500) in TBST for 1.5 h at 25℃. c-MYC protein was detected by the c-MYC antibody at working dilution 1:500 for 1 h at 25℃. The cells were washed with PBS three times and labeled with a secondary antibody (donkey anti-mouse IgG H&L (Alexa Fluor® 594); working dilution 1:500) in TBST for 1.5 h at 25℃. Finally, nuclei were stained with DAPI for 5 min. An inverted fluorescence microscope was used to capture images. All images and channels were placed in the same settings.

### Immunohistochemical staining

Sections of formalin-fixed, paraffin-embedded femoral arteries were dewaxed and rehydrated, and antigen retrieval was performed by incubating the sections for 10 min at 100°C in 10 mM citrate buffer (pH 6.0) followed by cooling at 25°C for 20 min. The sections were then incubated with hydrogen peroxide for 8 min at 25°C to quench the endogenous peroxidase. After antigen recovery, sections were incubated overnight at 4°C with a primary antibody. Sections were then washed with PBS and incubated with HRP-conjugated secondary antibodies at 25°C for 0.5 h. After PBS washing, the sections were incubated in DAB peroxidase substrate solution until the desired stain intensity was shown. Image-Pro Plus 6.0 professional image analysis software was used to measure protein expression.

### Statistical analysis

All data were presented as the mean ± SD of at least three separate experiments. For the comparisons between two groups, Student's t-test was used for independent samples. One-way analysis of variance (ANOVA) test with Dunnett's method was performed to compare multiple groups. *P <* 0.05 was considered statistically significant. GraphPad Prism version 8.0 software (GraphPad Software, Inc., La Jolla, CA, USA) was used for statistical analyses.

## Results

### Celastrol attenuated neointimal formation *in vivo*, and inhibited VSMCs proliferation and migration *in vitro*

**Figure 1:**
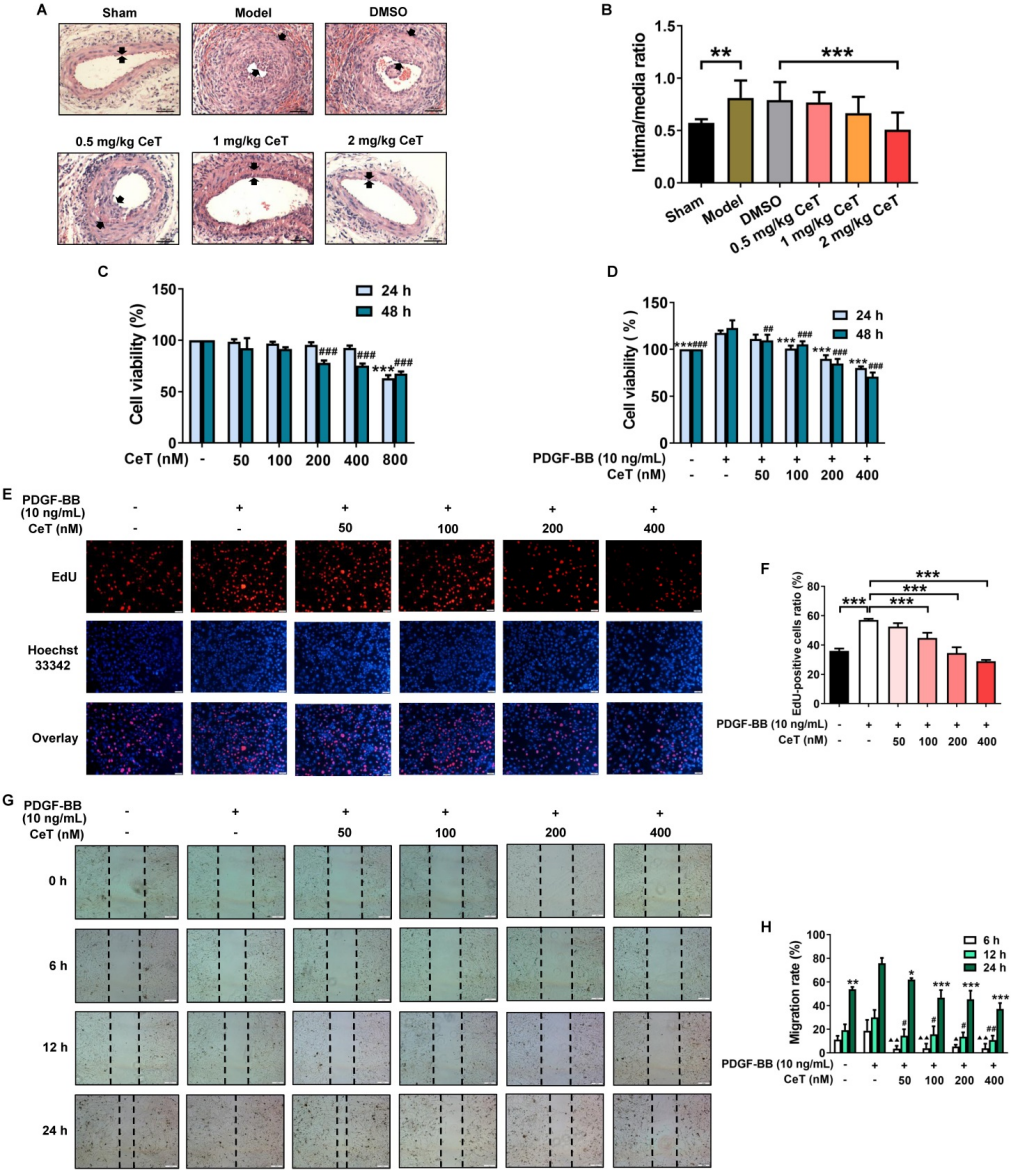
** Celastrol inhibited neointimal formation and VSMCs proliferation and migration.** (A) The cross-sectional HE images of femoral artery in the seven groups (200× of magnification, scale bar representing 100 μm). The area between the two arrows represents the thickest part of the intima. (B) The neointima/media ratio was used to quantify the vascular intimal hyperplasia of the femoral artery. ^**^P < 0.01, ^***^P < 0.001. (C) VSMCs were treated with 50-800 nM celastrol for 24 h or 48 h, and cell viability was evaluated by CCK-8 assay. ^###^*P <* 0.001 and ^***^P < 0.001 *versus* control group. (D) VSMCs were pre-exposed to 50-400 nM celastrol for 1 h, and then supplemented with 10 ng/mL PDGF-BB for 24 h or 48 h co-treatment. The cell viability was examined by CCK-8 assays. ^##^*P <* 0.01 , ^###^*P <* 0.001 and ^***^P < 0.001 *versus* PDGF-BB-stimulated group. (E) The cell viability was detected using EdU assays (magnification of ×100, scale bar representing 50 μm). (F) Relative ratio of EdU-positive cells. ^***^P < 0.001 *versus* PDGF-BB-stimulated group. (G) The wound healing images were analyzed using an inverted microscope (magnification of ×40, scale bar representing 200 μm). (H) The migration rate of VSMCs. ^▲^*P <* 0.05, ^▲▲^*P <* 0.01, ^#^*P <* 0.05, ^##^*P <* 0.01, ^*^P < 0.05, ^**^P < 0.01, and ^***^P < 0.001 *versus* PDGF-BB-stimulated group. All data were shown as the mean ± SD of three independent experiments.

The abnormal proliferation and migration of VSMCs promote a large amount of neointimal formation after vascular injury or occlusion 31, 32. The successful establishment of the mouse model of restenosis was confirmed by the intima/media ratio of the vessels. As shown in Figure 1A,B, compared with the sham operation group, the intima/media ratio of model group was dramatically increased. Administration of celastrol significantly reduced neointimal formation in the injured femoral artery in a dose-dependent manner, as demonstrated by the decrease in neointima/media ratio (Figure 1A,B). These data demonstrated that celastrol effectively attenuated neointimal hyperplasia.

The effect of celastrol *in vitro* was further investigated in VSMCs stimulated with PDGF-BB that is one of the most effective reagents to stimulate cell proliferation and migration 33. No obvious toxicity was detected in VSMCs with treatments of 50-400 nM celastrol for 24 h or 50-100 nM celastrol for 48 h (Figure 1C). However, we found that celastrol inhibited PDGF-BB-induced VSMCs proliferation in a dose- and time-dependent manner (Figure 1D-F). Celastrol at a concentration of 400 nM had the highest inhibitory effect on PDGF‐BB-induced proliferation of VSMCs (Figure 1D-F). Furthermore, celastrol significantly blocked the migration of VSMCs (Figure 1G,H).

### Celastrol activated VSMCs autophagy *in vivo* and *in vitro*

**Figure 2:**
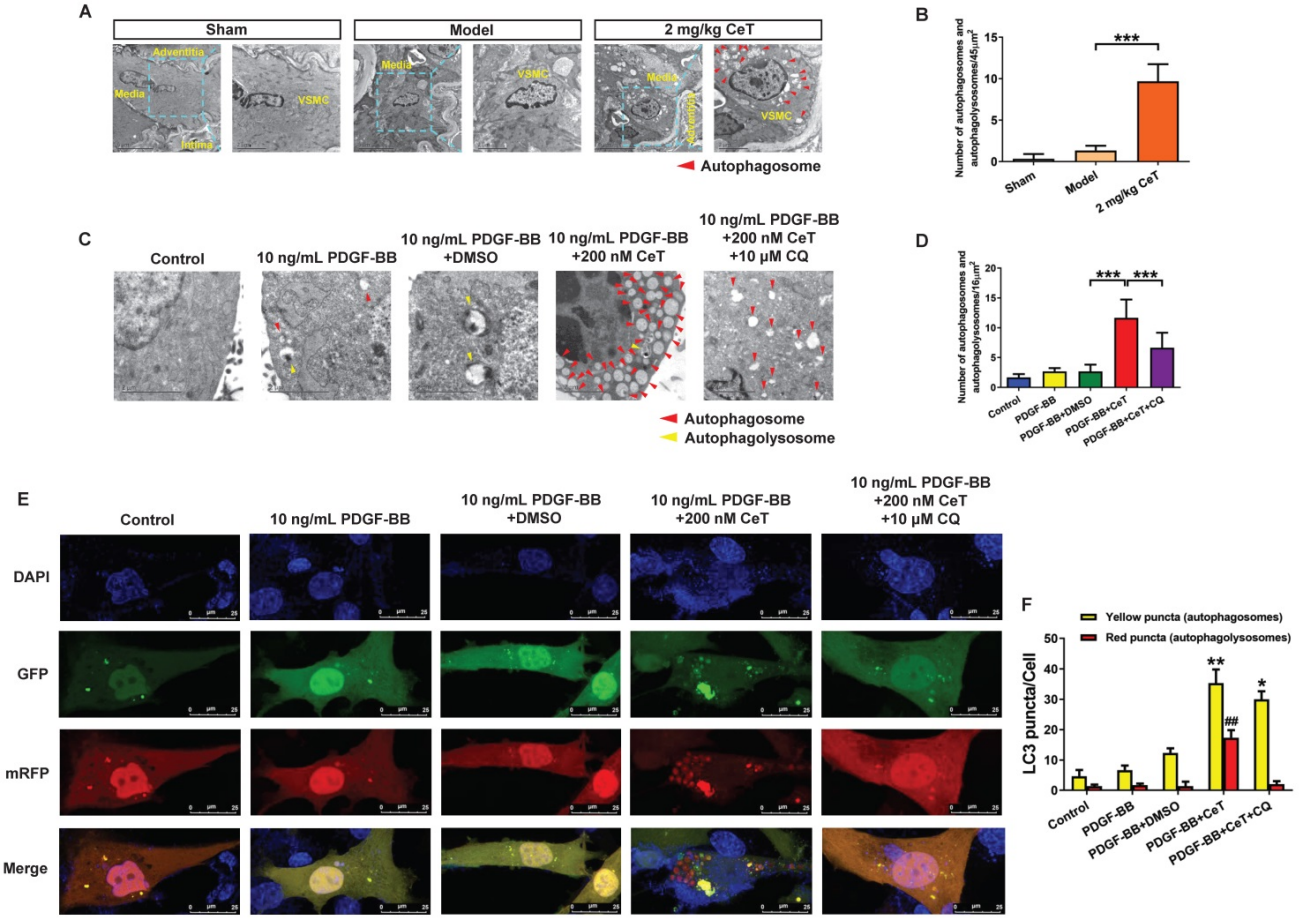
** Celastrol enhanced VSMCs autophagy *in vivo and in vitro*.** (A) The images of the ultrastructural features of autophagy (autophagosomes) in VSMCs from femoral artery were detected by TEM (scale bar representing 2 μm and 5 μm). (B) The bar graph showed the number of autophagic vacuoles per optical section. ^***^P < 0.001. VSMCs were treated with 200 nM celastrol in the presence or absence of 10 μM CQ for 24 h. (C) The images of intracellular autophagic vacuoles (autophagosomes and autophagolysosomes) were observed in VSMCs using TEM (scale bar representing 2 μm). (D) The number of autophagic vacuoles per optical section were analyzed. ^***^P < 0.001. (E) VSMCs were transfected with adenovirus harboring tandem fluorescent mRFP-GFP-LC3 and stained with DAPI (blue fluorescent). The yellow puncta (GFP/mRFP) represented the autophagosomes and the red puncta (mRFP) represented autophagolysosomes in the merged picture (scale bar representing 25 μm). (F) The number of autophagosomes or autophagolysosomes was counted per cell and quantified. Compared to PDGF-BB+DMSO group, ^##^*P <* 0.01, ^*^P < 0.05 and ^**^P < 0.01. The results were shown as the mean ± SD of three independent experiments.

The observation of autophagic vacuoles (including autophagosomes and autophagolysosomes) through TEM as the most direct and classic method for autophagy identification were examined in the present study. As shown in Figure 2A,B, compared with the model group, celastrol induced a significant accumulation of VSMCs autophagosomes in the injured femoral artery, indicating that celastrol could activate VSMCs autophagy *in vivo*.

Consistent with *in vivo* results, the number of autophagosomes and autophagolysosomes increased dramatically* in vitro* in celastrol-treated group (Figure 2C,D). However, the autophagic vacuolization was rarely observed by TEM *in vitro* in control, PDGF-BB-stimulated, and solvent-treated group (Figure 2C,D). CQ, as the autophagy inhibitor, can inhibit lysosomal activity and prevent the formation of autophagolysosomes 34. To determine the role of autophagy for this event, VSMCs were treated with a combination of celastrol and CQ. We observed that celastrol promoted autophagosomes formation, whereas CQ could reverse the formation of autophagolysosomes induced by celastrol (Figure 2C,D).

Generally, autophagy process mainly includes the formation of autophagosomes, fusion with lysosomes and degradation of lysosomes, *etc*. To elucidate the effects of celastrol on the autophagy process in VSMCs, double-labeled adenovirus emiting mRFP (red) and GFP (green) fluorescent signals was used to label LC3 and to detect the autophagic flux. Since GFP is sensitive to acidic environment, the fluorescence of GFP is quenched when autophagosomes and lysosomes fused, and only emission of the fluorescence of RFP can be observed. The simultaneous presence of GFP and RFP fluorescence (shown as yellow fluorescence) indicates that autophagosomes have not yet been fused to lysosomes. Here, a large number of yellow and red puncta were observed in the 200 nM celastrol treatment group (Figure 2E,F). However, few yellow and red spots were detected in the control group, PDGF-BB stimulation group and solvent treatment group (Figure 2E,F). In addition, in the presence of CQ, celastrol significantly increased multiple yellow puncta, but only few red puncta, indicating that celastrol-induced autophagic flux was impaired by CQ (Figure 2E,F). Overall, these results suggested that celastrol could activate VSMCs autophagy both *in vivo* and* in vitro*.

### Celastrol inhibited abnormal proliferation of VSMCs through autophagy-mediated c-MYC degradation

**Figure 3:**
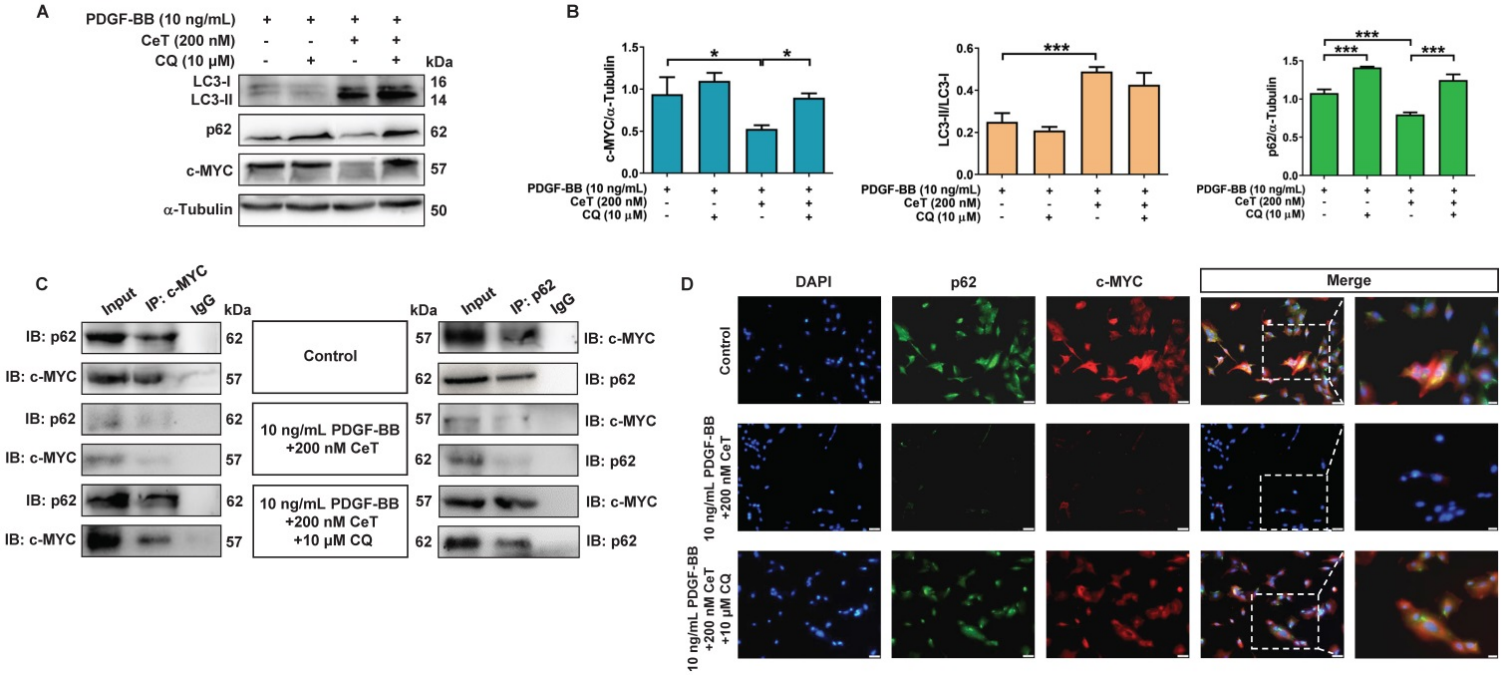
** Celastrol promoted autophagy to degrade c-MYC.** (A) The levels of LC3II/LC3I, p62, and c-MYC were detected by immunoblotting. (B) The expression of LC3II/LC3I, p62, and c-MYC was quantified by densitometric analysis. α-Tubulin was used as a loading control. ^*^P < 0.05 and ^***^P < 0.001. (C) The whole-cell lysates were immunoprecipitated with anti-c-MYC antibody or anti-p62 antibody and blotted with anti-p62 antibody or anti-c-MYC antibody. (D) Double immunofluorescence for p62 and c-MYC. The nuclei were stained with DAPI. The green channel showed the fluorescence signal of p62 antibody. The red channel showed the fluorescence signal of c-MYC antibody. Co-localization was yellow (magnification of ×100, scale bar representing 50 μm and magnification of ×200, scale bar representing 20 μm). All values were presented as the mean ± SD of three independent experiments.

LC3 and p62 are two key proteins during autophagy process 35. The conversion of LC3 from LC3-I (cytosolic form) to LC3-II (membrane-binding form) and autophagic degradation of p62 are considered to be effective markers of autophagy. Notably, autophagy acts as a negative regulator of c-MYC function 36. c-MYC belongs to a highly pleiotropic transcription factor that plays a crucial role in the growth and proliferation of VSMCs 37. In this study, celastrol increased LC3II/LC3I ratio while weakening the expression of p62 and c-MYC in VSMCs (Figure 3A,B). However, in the presence of CQ, celastrol treatment increased the ratio of LC3II/LC3I, whereas p62 and c-MYC levels did not decrease (Figure 3A,B), suggesting that CQ hindered the celastrol-induced fusion of autophagosomes and lysosome.

Next, the celastrol-induced autophagy was further evaluated in relation to c-MYC. Co-IP assays revealed that c-MYC was identified as a potential p62-binding partner *in vitro* (Figure 3C). Double-labeled immunofluorescence staining showed that the fluorescent p62 label was consistently localized with the fluorescent c-MYC label supported by a yellow fluorescent label (Figure 3D). In addition, celastrol downregulated the expressions of p62 and c-MYC *in vitro*, which was prevented by CQ (Figure 3C,D). These findings suggested that c-MYC could be transported to autophagosomes by binding to p62, and celastrol might inhibit VSMCs proliferation through autophagy-induced lysosomal degradation of c-MYC.

### Celastrol activated autophagy by Wnt5a/PKC/mTOR signaling pathway in VSMCs

**Figure 4:**
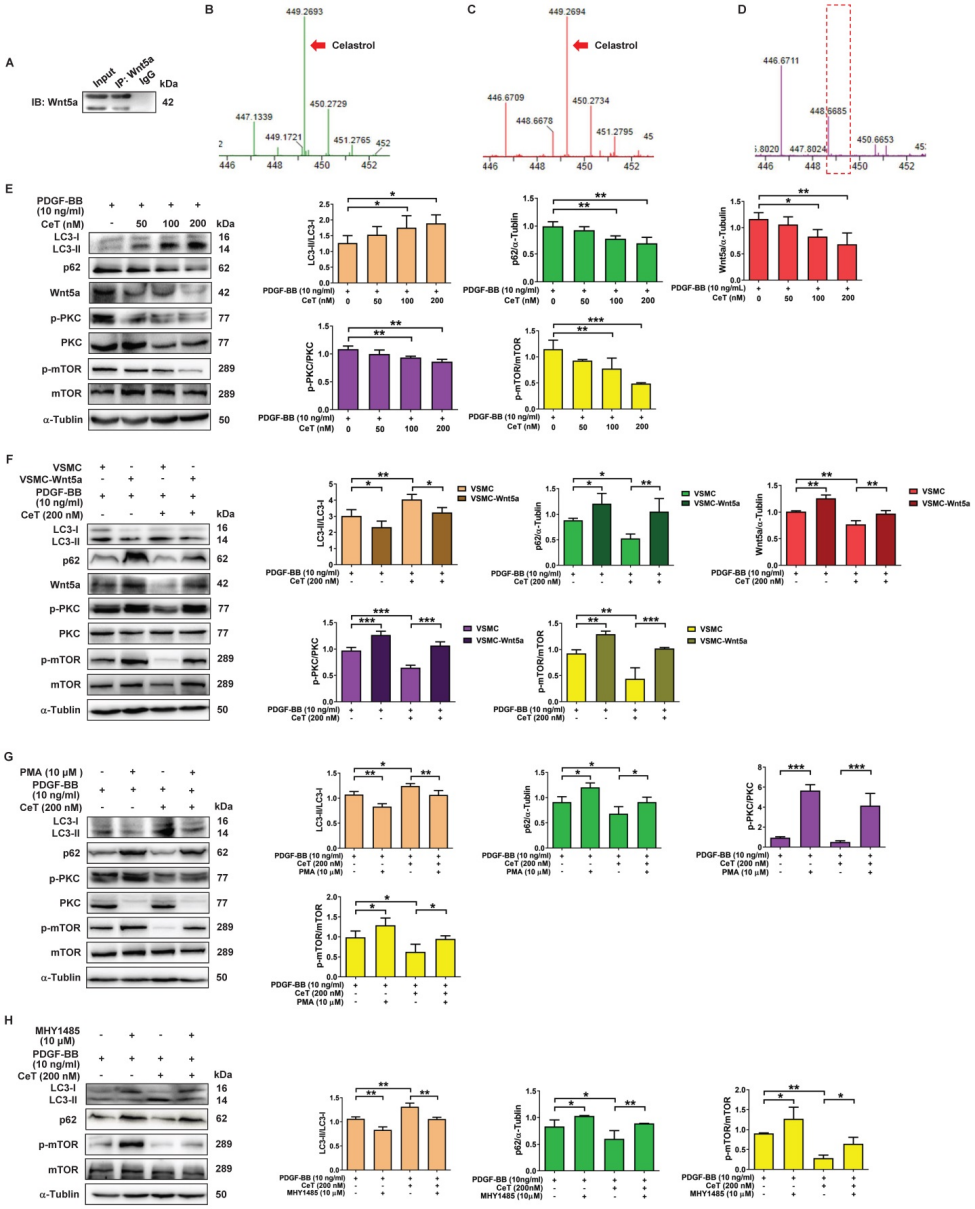
** Wnt5a/PKC/mTOR signaling pathway participated in celastrol-induced autophagy in VSMCs.** (A) Whole-cell lysates were immunoprecipitated and blotted with anti-Wnt5a antibody. (B) MS analysis of celastrol. (C) MS analysis of celastrol in the presence of Wnt5a protein. (D) MS analysis of celastrol without Wnt5a protein. (E) VSMCs were treated with 10 ng/mL PDGF-BB in the presence or absence of 50-200 nM celastrol for 24 h, and then the expression levels of LC3II/LC3I, p62, Wnt5a p-PKC, PKC, p-mTOR and mTOR were analyzed by western blot analysis, and was quantified by densitometric analysis using image laboratory software. α-Tubulin was used as a loading control. (F) VSMCs and VSMC-Wnt5a were incubated with 10 ng/ml PDGF-BB in the presence or absence of 200 nM celastrol for 24 h. Cell lysates were subjected to western blot analysis by using primary antibody specific for LC3II/LC3I, p62, Wnt5a, p-PKC, PKC, p-mTOR and mTOR. And the expression of LC3II/LC3I, p62, Wnt5a, p-PKC/PKC and p-mTOR/mTOR were quantified by densitometric analysis. α-Tubulin was used as a loading control. (G) VSMCs were treated with 10 ng/ml PDGF-BB in the presence or absence of 200 nM celastrol or 10 μM PMA for 24 h. The levels of LC3II/LC3I, p62, p-PKC, PKC, p-mTOR and mTOR were determined by western blot analysis, with α-Tubulin as a loading control. And bar graph showing the protein expressions of LC3II/LC3I, p62, p-PKC/PKC and p-mTOR/mTOR. (H) VSMCs were incubated with 10 ng/ml PDGF-BB in the presence or absence of 200 nM celastrol or 10 μM MHY-1485 for 24 h. The expression levels of LC3II/LC3I, p62, p-mTOR and mTOR were determined by western blot analysis with α-Tubulin as a loading control and was quantified by densitometric analysis. ^*^P < 0.05, ^**^P < 0.01 and ^***^P < 0.001. The data were represented as the mean ± SD of three independent experiments.

Wnt5a belongs to the secretory glycoprotein and is a member of the Wingless/integrase 1 (WNT) family. Celastrol is a quinone methide triterpenoid and contains electrophilic sites within the rings of quinone methide structure. The electrophilic sites can bind to nucleophilic thiol groups of cysteine residues to form covalent Michael adducts 38. Interestingly, Wnt5a has approximately 22 highly conserved cysteine residues 39. Furthermore, downregulation of Wnt5a may have an inhibitory effect on the proliferation of VSMCs 40. To address whether celastrol, as a Michael acceptor, might capture Wnt5a at its target binding site to form covalent adducts, celastrol with or without Wnt5a protein was immunoprecipitated with Wnt5a antibody, followed by MS analysis in the present study. MS identified the characteristic peaks of celastrol at mass-to-charge (m/z) 449.269 (Figure 4B). As shown in Figure 4A,C,D, in the presence of Wnt5a protein, MS detected a characteristic peak of celastrol at m/z 449.269, whereas in the absence of Wnt5a protein, it was no peak at m/z 449.269. The data demonstrated the interaction between celastrol and Wnt5a.

To investigate the potential mechanism of celastrol-induced VSMCs autophagy, we tried to find the molecular target of celastrol. As shown in Figure 4E, the ratio of LC3II/LC3I was remarkably increased, whereas the expression of p62, c-MYC, Wnt5a, the ratio of p-PKC/PKC and p-mTOR/mTOR were dramatically decreased in a dose-dependent manner after celastrol treatment. Next, we constructed a stable Wnt5a-overexpression VSMCs line (VSMC-Wnt5a) by lentivirus transfection, used the PKC activator PMA and utilized mTOR activator MHY-1485 to verify the molecular mechanism of celastrol-induced autophagy. The results showed that overexpression of Wnt5a, phosphorylation-dependent activation of PKC and mTOR could reverse the effect of celastrol on autophagy and the molecular targets in VSMCs, respectively (Figure 4F-H). Additionally, Wnt5a overexpression could promote activation of PKC and mTOR, and PKC activator PMA could stimulate mTOR phosphorylation (Figure 4F-H). Taken together, these results indicated that celastrol promoted VSMCs autophagy via the Wnt5a/PKC/mTOR signaling pathway.

### Celastrol induced VSMCs autophagy by Wnt5a/PKC/mTOR signaling pathway *in vivo*

**Figure 5:**
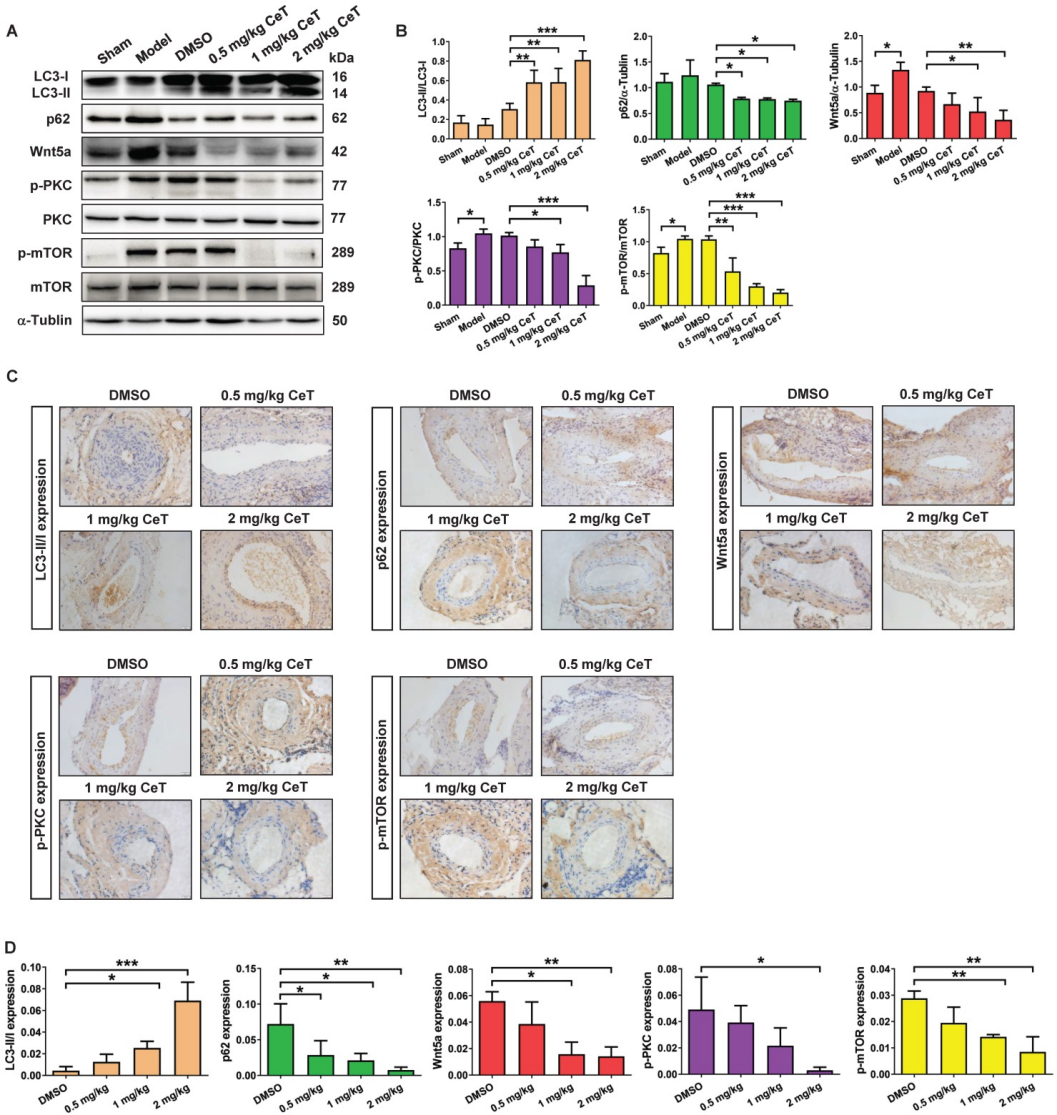
** Celastrol triggered VSMCs autophagy by Wnt5a/PKC/mTOR signaling pathway in femoral artery.** (A) Western blot was analyzed by using primary antibody specific for LC3II/LC3I, p62, Wnt5a, p-PKC, PKC, p-mTOR and mTOR with α-Tubulin as a loading control. (B) The histograms showed the quantification of LC3II/LC3I, p62, Wnt5a, p-PKC/PKC and p-mTOR/mTOR. (C) The immunohistochemical micrographs of the cross-sections of the femoral arteries (magnification of ×200, scale bar representing 20 μm). (D) Quantitative analysis of LC3II/LC3I, p62, Wnt5a, p-PKC and p-mTOR in the tissue of femoral artery through the image operating system. ^*^P < 0.05, ^**^P < 0.01 and ^***^P < 0.001. All values were presented as the mean ± SD of three independent experiments.

Then, we further evaluated whether celastrol inhibited neointimal formation by Wnt5a/PKC/mTOR signaling pathway in injured vessels. The results from western blot analysis and immunohistochemistry assays indicated that celastrol significantly increased LC3, and obviously decreased the expression of p62, Wnt5a, p-PKC, and p-mTOR in a dose-dependent manner in injured femoral arteries (Figure 5A-D). These data demonstrated that Wnt5a/PKC/mTOR signaling pathway were involved in celastrol-induced suppression of intimal hyperplasia.

### Knockdown of Wnt5a attenuated neointimal formation and induced VSMCs autophagy by PKC/mTOR signaling pathway *in vivo*

**Figure 6:**
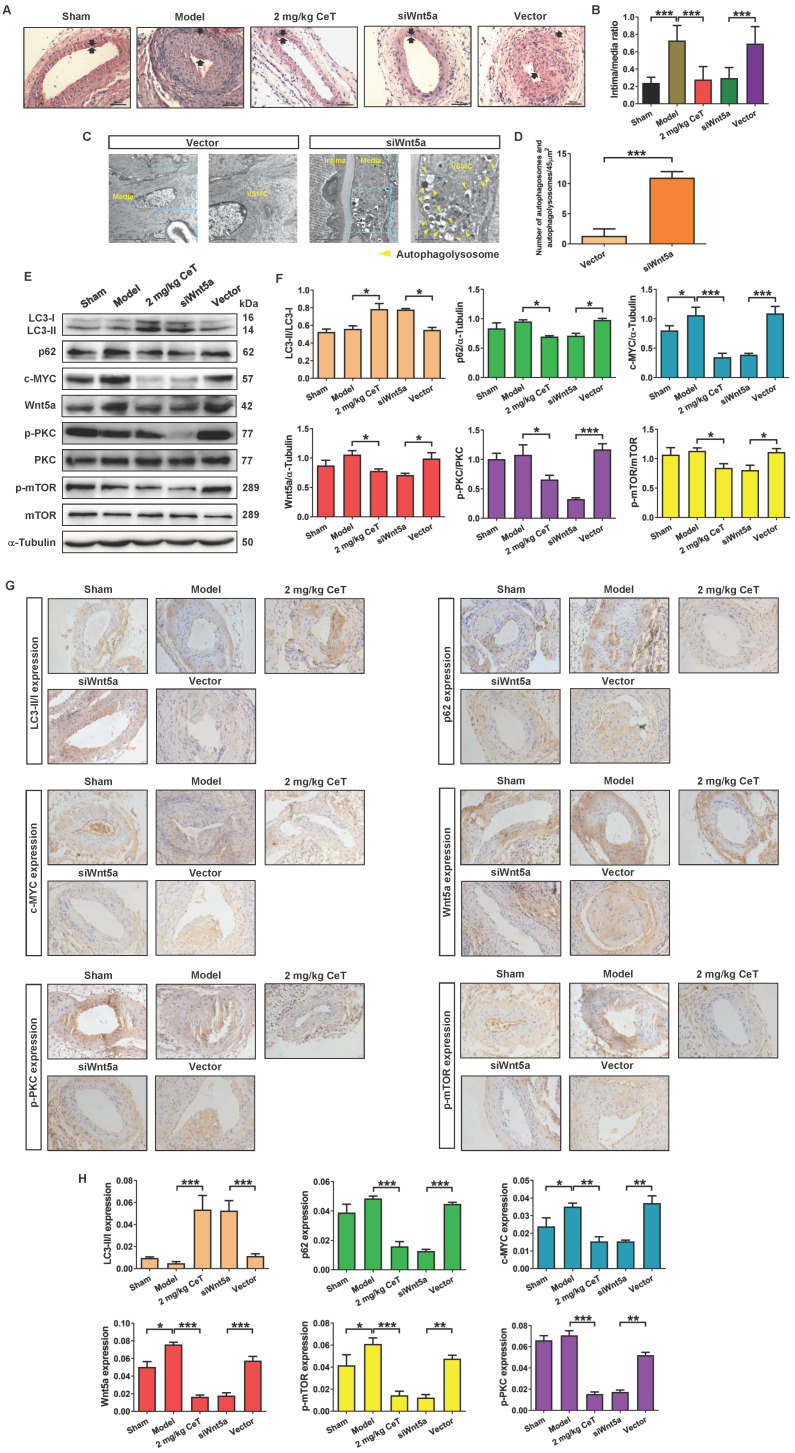
** The effect of Wnt5a knockdown on neointimal formation and autophagy of VSMCs *in vivo*.** (A) The HE staining images of mouse femoral artery in different treatment groups (magnification of ×200, scale bar representing 100 μm). The area between the two arrows represents the thickest part of the intima. (B) The formation of neointima in femoral artery was determined by neointima/media ratio. (C) Images of the ultrastructural feature of autophagy (autophagolysosomes) in VSMCs of the femoral artery, measured by TEM (scale bar representing 2 μm and 5 μm). (D) Histogram represented the number of autophagic vacuoles per optical section. (E) The levels of the LC3II/LC3I, p62, c-MYC, Wnt5a, p-PKC, PKC, p-mTOR and mTOR in femoral artery were analyzed by western blot analysis, with α-Tubulin as a loading control. (F) The densitometry analysis of LC3II/LC3I, p62, c-MYC, Wnt5a, p-PKC/PKC and p-mTOR/mTOR. (G) The image of immunohistochemistry for cross-sections of the femoral artery (magnification of ×200, scale bar representing 20 μm). (H) Quantitative analysis of LC3II/LC3I, p62, c-MYC, Wnt5a, p-PKC and p-mTOR in femoral artery tissues using an image manipulation system. ^*^P < 0.05, ^**^P < 0.01 and ^***^P < 0.001. Data were shown as the mean ± SD of three independent experiments.

To verify whether celastrol reduced VSMCs proliferation and neointimal formation by down-regulating Wnt5a, adenovirus-mediated small interfering RNA (siRNA) was applied in this study. Two weeks after femoral arterial injury, neointimal formation was clearly observed in the model group (Figure 6A,B). However, compared with the adenovirus vector group or model group, knockdown of Wnt5a by using siWnt5a or celastrol treatment significantly reduced the intima/media ratio (Figure 6A,B). Thus, knockdown of Wnt5a had an effect similar to celastrol in inhibiting neointimal hyperplasia.

We next investigated whether Wnt5a knockdown was involved in VSMCs autophagy. As shown in Figure 6C,D, compared with the adenovirus vector group, siWnt5a treatment significantly increased the number of autophagosomes and autophagolysosomes in VSMCs of femoral artery. These data indicated that knockdown of Wnt5a significantly activated VSMCs autophagy in the injured femoral artery.

Meanwhile, either celastrol or Wnt5a knockdown could up-regulated the ratio of LC3II/LC3I, while significantly down-regulated the expressions of p62, c-MYC, Wnt5a, p-PKC, and p-mTOR (Figure 6E-H). These results suggested that celastrol could induce autophagy to degrade c-MYC by preventing Wnt5a/PKC/mTOR signaling pathway.

## Discussion

In the present study, we observed the protective effects of celastrol on neointimal formation and VSMCs proliferation. The direct target of celastrol was identified and the possible mechanisms by which celastrol protects blood vessels from restenosis was suggested through activating VSMCs autophagy. It seems that celastrol-induced autophagy promoted the lysosomal degradation of p62 and c-MYC conjugates, thereby inhibiting the proliferation of VSMCs. In our previous study, we have found that the expression of Wnt5a is notably up-regulated in both patients with atherosclerosis and apoE^-/-^ mice 30. Consistent with this, the model group with neointimal formation in the present study showed a high level of Wnt5a. Importantly, we found that Wnt5a/PKC/mTOR signaling pathway was involved in celastrol-induced autophagy and inhibition of VSMCs proliferation and neointimal formation.

The abnormal proliferation of VSMCs and the formation of intimal hyperplasia often result in vascular occlusive diseases, such as restenosis and transplant vasculopathy 41, 42. Among the three arterial layers, the media is mainly composed of VSMCs. Fully mature VSMCs possess an inherently extensive plasticity, and are usually in a non-proliferative and contractile state 43. After vascular injury, differentiated VSMCs in the medial layer convert from the contractile phenotype to the proliferative phenotype, and then migrate to the intima and secrete a large amount of extracellular matrix to form neointima 44, 45. Therefore, alleviation of the proliferation and migration of VSMCs is crucial for the treatment of intimal proliferative diseases after vascular injury. The mouse model of femoral artery injury is a well-defined and recognized model of neointimal formation and restenosis. In addition to the *in vivo* model, we also established an *in vitro* model of VSMCs proliferation stimulated by PDGF-BB for the present study. In both *in vivo* and* in vitro* experiments, we noted that celastrol could reduce neointimal formation and VSMCs proliferation. Interestingly, autophagy was involved in the inhibition of VSMCs proliferation and neointimal hyperplasia induced by celastrol.

Autophagy is an evolutionarily conserved process that can engulf excess, long-lived or damaged cytoplasmic material and delivered to lysosomes for degradation.^36^ Several studies have revealed that autophagic activity directly or indirectly determines the phenotypic transformation and viability of VSMCs during the progression of vascular diseases 46, 47. More importantly, the activation of autophagy significantly inhibits the proliferation of VSMCs. Free fatty acids, such as saturated palmitic acid and unsaturated oleic acid, enhance autophagy flux and then suppress VSMCs growth 48. In addition, the mTOR pathway can regulate autophagy and proliferation of VSMCs under normal or hypoxic conditions 49. Meanwhile, the activation of TFEB-mediated autophagy hinders VSMC dedifferentiation, leads to a decrease in synthetic phenotype conversion, and prevents high-fat diet-induced intimal hyperplasia 13. However, the mechanism by which autophagy inhibits the proliferation of VSMCs remains controversial. In our study, celastrol could induce autophagy, which is manifested by activating autophagosomes and autophagolysosomes, enhancing autophagy flux, and increasing the conversion of LC3-I to LC3-II and p62 degradation *in vivo* and* in vitro*. As expected, we showed that the inhibition of autophagy by CQ could reverse the anti-proliferative effect mediated by celastrol in VSMCs. In addition, we found that c-MYC played a critical role in celastrol-induced inhibition of VSMCs proliferation.

**Figure 7:**
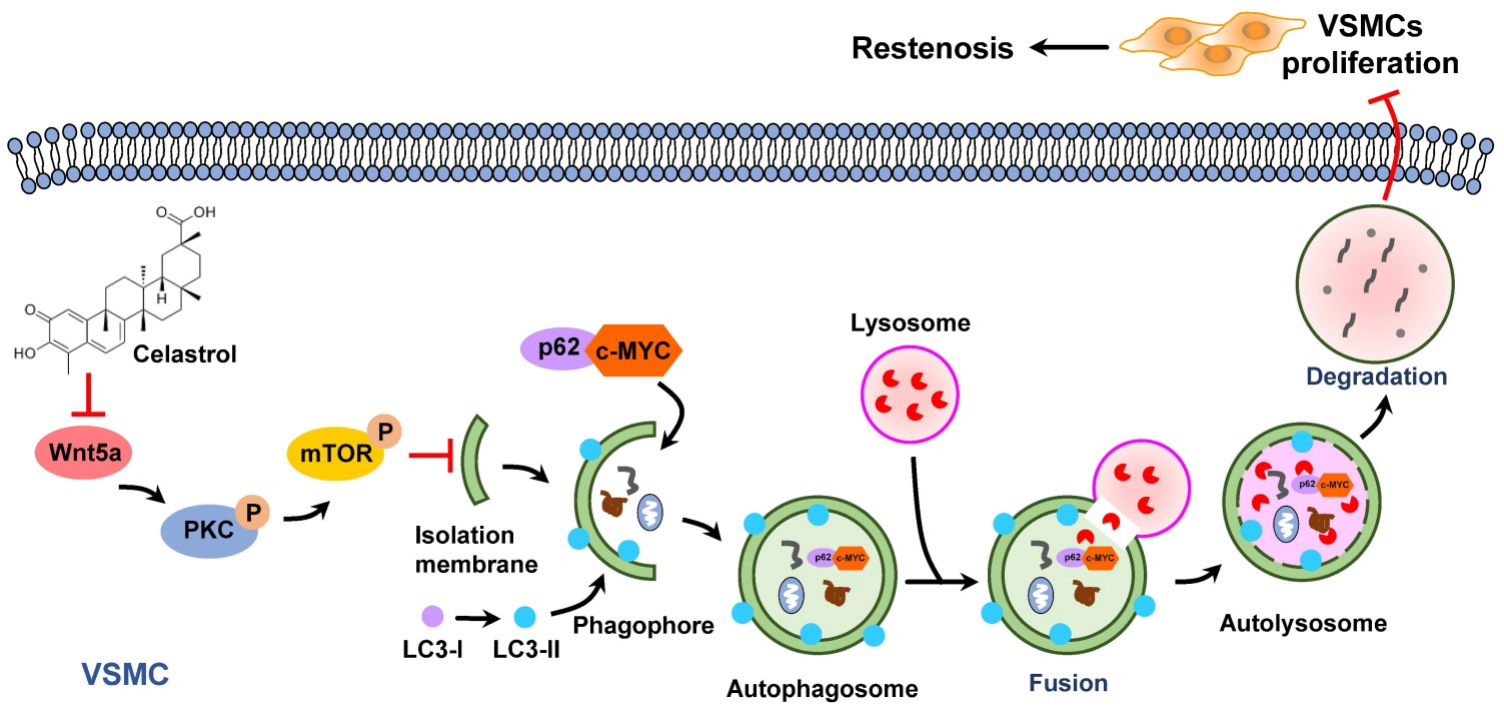
** Graphical summary illustrating the inhibitory effect of celastrol on restenosis.** Celastrol promotes autophagy-induced degradation of complexes of c-MYC and p62 by impeding Wnt5a/PKC/mTOR signaling pathway, thereby inhibiting abnormal VSMCs proliferation and vascular restenosis.

Previous studies have shown that increased expression of c-MYC protein is closely associated with certain cardiovascular diseases, such as atherosclerosis, hypertension and restenosis 50. c-MYC belongs to the basic helix-loop-helix leucine zipper (bHLHZip) family of transcription factors and can induce the continuous proliferation of VSMCs 51-53. In this study, we demonstrated that inhibition of c-MYC expression was a key point for the anti-proliferative effect of celastrol in VSMCs. In the process of autophagy, p62 acts as an autophagic cargo protein that binds endogenous autophagy substrates to autophagolysosome degradation 54. Most importantly, we demonstrated that c-MYC and p62 combined to form a conjugate, which was then degraded *via* the autophagy-lysosome pathway. These studies elegantly supported a novel concept that c-MYC was delivered to lysosomes for degradation by interacting with p62 during the process of celastrol-induced VSMCs autophagy (Figure 7).

Wnt5a-involved autophagy was suggested as the possible mechanism of protective role of celastrol against vascular restenosis. Wnt5a can mediate a variety of cellular processes such as cell proliferation, migration, differentiation, and polarity during embryogenesis and development 55, 56. Previous study has reported that overexpression or knockdown of Wnt5a significantly up-regulates or down-regulates c-MYC expression 57. In addition, Wnt5a signaling regulates autophagy in therapeutic interventions 58, 59. Considering the important role of Wnt5a in regulating autophagy and c-MYC protein, we supposed that Wnt5a was a hub between celastrol and VSMCs autophagy, as well as VSMCs proliferation. Here, we found that Wnt5a was a potential target of celastrol in VSMCs through Co-IP and MS analysis. Intriguingly, celastrol could significantly reduce the expression of Wnt5a in VSMCs. Meanwhile, knockdown of Wnt5a activated autophagy, inhibited c-MYC expression and suppressed intimal hyperplasia. As a suggested molecular target of celastrol, Wnt5a was involved in the inhibition of excessive VSMCs proliferation.

Protein kinase C (PKC) is a family of serine/threonine kinases that have diverse cellular functions, including regulating cellular migration, proliferation and differentiation, etc 60, 61. A previous study showed that Wnt5a/PKC signaling can increase the capacity of proliferation, migration, invasion, and colony formation but reduce cell apoptosis in lung cancer cells 62. Activation of PKC signaling pathway enhances the proliferation of VSMCs, but the inhibition of PKC can reduce the growth VSMCs 63-65. mTOR is an atypical serine/threonine protein kinase and plays as a negative regulator in the process of autophagy 66, 67. PKC can regulate autophagy *via* mTOR pathway 68, 69. In addition, mTOR phosphorylation can regulate VSMCs migration and proliferation in atherosclerosis and restenosis 70, 71. The present study found that celastrol or knockdown of Wnt5a could attenuate phosphorylation-dependent activation of PKC and mTOR. However, Wnt5a overexpression, PKC activator PMA and mTOR activator MHY-1485 could reverse the effect of celastrol on autophagy and the molecular targets in VSMCs. Moreover, overexpression of Wnt5a could induce phosphorylation-dependent activation of PKC and mTOR, and PKC activation could promote mTOR phosphorylation. It seems reasonable to believe that celastrol activated autophagy by suppression of Wnt5a/PKC/mTOR signaling pathway, thereby promoting the lysosomal degradation of p62-c-MYC complexes in VSMCs.

In conclusion, as an active ingredient derived from traditional Chinese medicine *Tripterygium wilfordii*, celastrol has a powerful anti-intimal hyperplasia effect. The data have proved that celastrol promotes the autophagic degradation of c-MYC and p62 complexes by blocking the Wnt5a/PKC/mTOR signaling pathway, thereby inhibiting abnormal VSMCs proliferation and neointimal formation (Figure 7). The findings of the present study suggest a new drug and provide new therapeutic strategies to protect blood vessels from post-injury restenosis.
